# Low apolipoprotein B and LDL-cholesterol are associated with the risk of cardiovascular and all-cause mortality: a prospective cohort

**DOI:** 10.1080/07853890.2025.2529565

**Published:** 2025-07-11

**Authors:** Xiaolin Yu, Yujuan Yuan, Xiangyu Dong, Zulipiyemu Xier, Ling Ma, Hui Peng, Guoqing Li, Yining Yang

**Affiliations:** ^a^ Department of Cardiology, People’s Hospital of Xinjiang Uyghur Autonomous Region, Urumqi, China; ^b^ Xinjiang Key Laboratory of Cardiovascular Homeostasis and Regeneration Research, Urumqi, China

**Keywords:** Apolipoprotein B, LDL-cholesterol, all-cause mortality, cardiovascular mortality

## Abstract

**Background:**

The association between low-density lipoprotein (LDL) cholesterol and increased mortality risk has been well-documented, yet apolipoprotein B (apoB) is regarded as a more precise risk indicator. However, a comprehensive analysis integrating both markers in relation to mortality risk remains unreported.

**Objectives:**

This study aimed to investigate the relationship between LDL cholesterol levels and mortality across varying apoB concentrations within the general population.

**Methods:**

Data from 15,380 participants in the 2005–2016 National Health and Nutrition Examination Survey (NHANES) were utilized to construct Cox regression models and apply restricted cubic splines, assessing the association between LDL cholesterol and mortality across distinct apoB stratifications.

**Results:**

The study cohort had a median (IQR) age of 46.0 (32.0, 60.0) years, with 7949 (51.8%) males. During a median follow-up of 101.0 months (IQR: 67–137), 1771 (8.8%) all-cause mortality events were observed; 443 (2.1%) deaths were attributed to cardiovascular diseases, while 109 (0.5%) resulted from cerebrovascular diseases. Low apoB and LDL-cholesterol levels were independently linked to an elevated risk of all-cause and cardiovascular mortality. Compared with participants having apoB <90 mg/dL and LDL-cholesterol levels between 100–129 mg/dL, those with LDL-cholesterol <70 mg/dL (HR, 1.81; 95%CI: 1.39–2.36) and 70–99 mg/dL (HR, 1.28; 95%CI: 1.01–1.62) demonstrated a higher risk of all-cause mortality. Additionally, reduced apoB levels contributed to an increased risk of cardiovascular mortality among individuals with low LDL-cholesterol levels.

**Conclusions:**

Low apoB and LDL-cholesterol levels were associated with heightened all-cause and cardiovascular mortality risk in the general population. Conversely, high apoB and low LDL-cholesterol levels did not correlate with increased mortality risk.

## Introduction

Dyslipidemias, particularly hypercholesterolemia, represent primary risk factors for cardiovascular disease development and related mortality [1]. Low-density lipoprotein (LDL)-cholesterol serves as the principal target in lipid-lowering therapies for managing hypercholesterolemia and chronic coronary conditions [2–6]. A significant body of randomized controlled trials has demonstrated that LDL-cholesterol reduction lowers both total and cardiovascular mortality risk [7–9]. Nonetheless, some studies have shown an inverse relationship between LDL-cholesterol levels and all-cause mortality in certain patient populations [10–12]. The correlation between LDL-cholesterol and cardiovascular mortality in the general population remains inconsistent. While some observational studies indicate a positive correlation [13,14], others suggest a U-shaped association [15]. Furthermore, evidence suggests that low LDL-cholesterol levels may substantially increase the risk of intracerebral hemorrhage (ICH) and cancer in healthy individuals [16,17]. This highlights the need for further investigation into the relationship between low LDL-cholesterol and both all-cause and cause-specific mortality risks across diverse populations.

Apolipoprotein B (apoB) is a key structural component of atherogenic lipoproteins, which include all circulating lipoprotein particles except high-density lipoproteins (HDLs). As a measure of the total number of atherogenic particles, apoB reflects the atherogenicity of both cholesterol-rich and cholesterol-depleted lipoproteins. ApoB and LDL-cholesterol are distinct yet strongly correlated parameters [18,19]. Compared to LDL-cholesterol, ApoB serves as a more precise indicator of mortality risk [20–23]; however, a conventional analysis within the UK Biobank did not corroborate these results [24]. Mendelian randomization has suggested that the clinical benefits of reducing LDL-cholesterol may be contingent upon the corresponding decrease in apoB-containing lipoproteins [25]. Cole J et al. observed that 40% of general population with very low LDL-cholesterol exhibited disproportionately high apoB levels, while 25%–40% of individuals demonstrated concordant levels of both parameters [26]. This highlights the widespread occurrence of discordance between ApoB and LDL-cholesterol levels.

To our knowledge, the relationship between low LDL-cholesterol levels and mortality in the general population across varying apoB levels remains unexplored. This study aimed to investigate the potential association between LDL-cholesterol and mortality, stratified by different apoB levels, in the general population. These findings may help refine risk assessment and guide personalized lipid-lowering strategies in clinical practice, while also informing future research on the complex interplay between LDL-cholesterol, apoB, and health outcomes.

## Materials and methods

### Study population and study design

**Figure 1.**
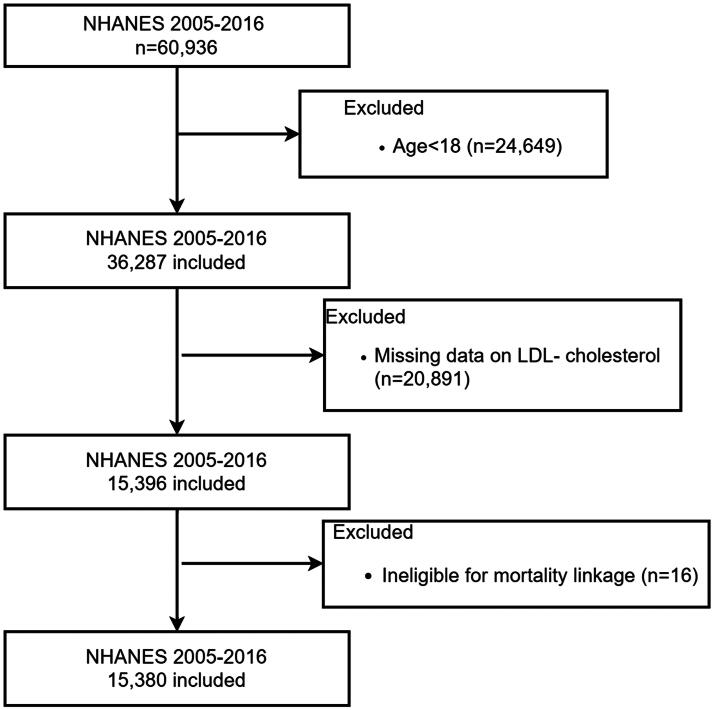
Flowchart of the study design.

The National Health and Nutrition Examination Survey (NHANES) database serves as a continuous research initiative aimed at evaluating the health and nutritional status of adults and children in the United States. This study analyzed data from six NHANES cycles spanning 2005–2016, focusing on adult participants with available apoB and LDL-C measurements. Among the 60,936 individuals surveyed during this period, 36,287 were aged 18 years or older. After excluding 20,891 individuals lacking LDL-cholesterol data, the analytical cohort comprised 15,396 participants. An additional 16 individuals were removed due to missing survival status, yielding a final sample of 15,380 participants (Figure 1).

### Exposure and clinical outcomes

Blood lipid parameters, including total cholesterol, triglycerides, HDL-cholesterol, and apoB, were documented on the NHANES website. Distribution estimates were restricted to participants aged ≥12 who had undergone fasting for 8.5–24 h. ApoB concentrations in serum samples were quantified *via* immunochemical assays. LDL-cholesterol levels were derived from total cholesterol, triglycerides, and HDL-cholesterol values using the Friedewald equation [27]: [LDL-cholesterol] = [total cholesterol] − [HDL-cholesterol] − [triglycerides/5], where [triglycerides/5] is an estimate of VLDL-cholesterol. All concentrations were reported in mg/dL, and the equation was applicable for triglyceride levels below 400 mg/dL.

The primary outcome was all-cause mortality, while cardiovascular and cerebrovascular mortality constituted secondary outcomes. Follow-up mortality data were sourced from NCHS mortality files, with participant outcomes documented through December 31, 2019.

### Covariates

Age, sex, race (Mexican American, other Hispanic, non-Hispanic White, non-Hispanic Black, and other race), marital status (widowed, divorced, separated, never married, and living with a partner), and education level (<9th grade, 9th–11th grade, high school graduate/GED or equivalent, some college or AA degree, and college graduate or above) were collected through participant interviews. The dataset also includes additional anthropometric and health metrics, such as height, blood pressure, body mass index (BMI), and waist circumference, which were collected as part of the standardized examination procedures. Smoking status was classified into never, current, and former smokers. Alcohol consumption was categorized as never, light, moderate, or heavy, based on a daily intake of 0, <1, 1–<8, and >8 drinks per week, respectively. Comorbidities included diabetes, hypertension, heart failure, coronary heart disease (CHD), stroke, emphysema, chronic bronchitis, and self-reported cancer. Individuals identifying as ‘borderline’ diabetic or ‘prediabetic’ were classified as non-diabetic. Laboratory assessments included triglycerides, total cholesterol, LDL-cholesterol, HDL-cholesterol, apoB, and lipid-lowering medication usage. Medications were grouped into statins (atorvastatin, fluvastatin, lovastatin, pravastatin, rosuvastatin, and simvastatin), non-statin cholesterol-lowering agents (ezetimibe, cholestyramine, probucol, colesevelam, and colestipol), and triglyceride-lowering therapies (fenofibrate, gemfibrozil, and niacin).

### Statistical analysis

Statistical analyses were conducted using R version 4.2.1. Given the complex survey design of NHANES, appropriate weighting ensured population-level representativeness. Continuous variables were summarized as weighted medians with interquartile ranges (IQR) due to their non-normal distribution, as determined by statistical tests, whereas categorical variables were expressed as weighted frequency percentages. Group comparisons were performed using the Wilcoxon rank-sum test for continuous variables and the chi-square test for categorical variables. Cox proportional hazards regression models were employed to estimate the associations between apoB and LDL-cholesterol levels with all-cause, cardiovascular, and cerebrovascular mortality. Time since cohort entry served as the underlying timescale, with censoring applied at the end of follow-up on December 31, 2019. Restricted cubic splines evaluated the dose-response relationships between apoB, LDL-cholesterol, and mortality outcomes. Three models were constructed: the Crude model, Model 1, and Model 2. Model 1 adjusted for age and sex, while Model 2 incorporated all covariates from Model 1 along with body mass index (BMI), race, marital status, education level, smoking and alcohol consumption status, systolic and diastolic blood pressure, and medical history of diabetes, hypertension, heart failure, CHD, stroke, emphysema, chronic bronchitis, and cancer. Additionally, Model 2 accounted for the use of statins, other cholesterol-lowering agents, and triglyceride-lowering medications. A *P* value <0.05 was considered statistically significant.

## Results

### Baseline characteristics of study participants

A total of 15,380 participants were included, with a median (IQR) age of 46.0 (32.0, 60.0) years; 7949 (51.8%) were male, and 6606 (58.1%) were non-Hispanic White. The weighted demographic baseline characteristics of participants are presented in Table 1. The median follow-up duration was 8.4 years, during which 1771 (8.8%) deaths were recorded. Participants were categorized into eight groups based on baseline LDL-cholesterol levels (<70, 70–99, 100–129, and ≥130 mg/dL) [28,29] and median apoB concentrations (<90 and ≥90 mg/dL) [22].

**Table 1.** Weighted baseline characteristics according to values of ApoB versus LDL cholesterol.

Variables		ApoB < 90mg/dL				ApoB ≥ 90mg/dL				*P* value*
	Overall, *N* = 15380	LDL-*C* < 70 mg/dL, *N* = 1467 (8.6%)	LDL-C: 70–99 mg/dL, *N* = 3950 (25%)	LDL-C: 100–129 mg/dL, *N* = 2121 (15%)	LDL-*C* ≥ 130 mg/dL, *N* = 122 (1.0%)	LDL-*C* < 70 mg/dL, *N* = 23 (0.1%)	LDL-C: 70–99 mg/dL, *N* = 461 (3.0%)	LDL-C: 100–129 mg/dL, *N* = 2821 (18%)	LDL-*C* ≥ 130 mg/dL, *N* = 4415 (29%)	
Sex										<0.001
Male	7,949.0 (51.8%)	706.0 (48.6%)	2,109.0 (54.2%)	1,206.0 (57.5%)	78.0 (57.1%)	14.0 (54.7%)	203.0 (47.3%)	1,383.0 (46.9%)	2,250.0 (51.3%)	
Female	7,431.0 (48.2%)	761.0 (51.4%)	1,841.0 (45.8%)	915.0 (42.5%)	44.0 (42.9%)	9.0 (45.3%)	258.0 (52.7%)	1,438.0 (53.1%)	2,165.0 (48.7%)	
Age (years)	46.0 (32.0, 60.0)	45.0 (26.0, 66.0)	41.0 (27.0, 60.0)	44.0 (31.0, 56.0)	46.0 (31.2, 58.0)	51.8 (34.0, 64.2)	52.0 (38.0, 65.0)	48.0 (36.0, 61.0)	49.0 (38.0, 59.0)	<0.001
Race										<0.001
Mexican American	2,540.0 (8.5%)	208.0 (7.4%)	620.0 (8.4%)	283.0 (7.2%)	11.0 (3.9%)	5.0 (5.8%)	91.0 (8.9%)	563.0 (10.1%)	759.0 (8.7%)	
Other Hispanic	1,535.0 (5.5%)	122.0 (5.0%)	350.0 (4.9%)	203.0 (5.5%)	7.0 (2.9%)	1.0 (3.2%)	42.0 (4.7%)	300.0 (5.9%)	510.0 (6.1%)	
Non-Hispanic White	6,606.0 (68.1%)	621.0 (65.6%)	1,665.0 (67.2%)	920.0 (68.4%)	73.0 (79.9%)	12.0 (80.6%)	226.0 (72.6%)	1,220.0 (68.0%)	1,869.0 (68.6%)	
Non-Hispanic Black	3,181.0 (10.8%)	372.0 (14.2%)	887.0 (11.8%)	478.0 (11.1%)	17.0 (6.3%)	3.0 (5.5%)	60.0 (6.2%)	488.0 (9.2%)	876.0 (10.3%)	
Other race	1,518.0 (7.1%)	144.0 (7.8%)	428.0 (7.7%)	237.0 (7.8%)	14.0 (7.0%)	2.0 (4.9%)	42.0 (7.6%)	250.0 (6.8%)	401.0 (6.2%)	
Married status										<0.001
Married	7,626.0 (54.9%)	625.0 (48.8%)	1,767.0 (49.9%)	991.0 (52.4%)	58.0 (52.2%)	9.0 (42.6%)	247.0 (58.6%)	1,498.0 (57.7%)	2,431.0 (60.1%)	
Widowed	1,178.0 (5.7%)	131.0 (7.0%)	279.0 (5.4%)	129.0 (4.2%)	12.0 (7.3%)	2.0 (15.5%)	40.0 (6.2%)	239.0 (6.0%)	346.0 (6.1%)	
Divorced	1,559.0 (10.0%)	122.0 (8.3%)	344.0 (8.3%)	223.0 (10.8%)	14.0 (11.8%)	2.0 (8.1%)	46.0 (8.9%)	285.0 (9.9%)	523.0 (11.6%)	
Separated	499.0 (2.2%)	44.0 (2.1%)	119.0 (2.0%)	72.0 (2.4%)	2.0 (0.9%)	1.0 (0.9%)	17.0 (1.9%)	88.0 (2.5%)	156.0 (2.3%)	
Never married	3,263.0 (19.1%)	433.0 (25.8%)	1,094.0 (25.0%)	521.0 (21.6%)	26.0 (19.6%)	6.0 (23.9%)	67.0 (16.9%)	479.0 (16.0%)	637.0 (13.0%)	
Living with partner	1,255.0 (8.1%)	112.0 (8.0%)	347.0 (9.4%)	185.0 (8.5%)	10.0 (8.2%)	3.0 (9.0%)	44.0 (7.5%)	232.0 (7.9%)	322.0 (6.9%)	
Education										0.005
Less than 9th grade	1,844.0 (6.5%)	189.0 (7.4%)	464.0 (6.8%)	212.0 (5.3%)	9.0 (4.1%)	4.0 (8.5%)	61.0 (5.8%)	360.0 (6.3%)	545.0 (6.8%)	
9–11th grade	2,342.0 (11.5%)	225.0 (12.4%)	607.0 (11.3%)	304.0 (10.6%)	13.0 (8.6%)	5.0 (23.4%)	89.0 (14.0%)	448.0 (12.3%)	651.0 (11.3%)	
High school graduate/GED or equivalent	3,431.0 (22.3%)	328.0 (22.3%)	803.0 (20.0%)	458.0 (20.9%)	17.0 (13.0%)	7.0 (35.4%)	113.0 (25.7%)	651.0 (23.4%)	1,054.0 (24.2%)	
Some college or AA degree	4,273.0 (30.6%)	408.0 (30.6%)	1,113.0 (31.0%)	587.0 (30.4%)	44.0 (41.9%)	4.0 (19.2%)	118.0 (28.5%)	773.0 (30.2%)	1,226.0 (30.4%)	
College graduate or above	3,490.0 (29.1%)	317.0 (27.4%)	963.0 (30.9%)	560.0 (32.9%)	39.0 (32.5%)	3.0 (13.6%)	80.0 (26.0%)	589.0 (27.8%)	939.0 (27.3%)	
Weight (kg)	79.30 (66.70, 93.90)	75.80 (63.30, 92.10)	75.80 (63.30, 91.00)	76.20 (64.65, 91.10)	81.22 (65.99, 94.44)	92.47 (71.75, 101.89)	88.60 (76.30, 102.00)	83.40 (70.60, 98.90)	81.10 (69.60, 94.50)	<0.001
Height (cm)	168.50 (161.60, 176.40)	169.30 (162.22, 176.60)	168.30 (161.60, 175.90)	168.14 (161.90, 176.24)	169.50 (161.85, 178.43)	164.72 (159.45, 173.35)	168.80 (160.80, 176.60)	169.30 (161.60, 177.00)	168.10 (161.00, 176.30)	0.140
BMI (kg/㎡)	27.60 (23.95, 32.20)	26.55 (22.70, 31.49)	26.28 (22.73, 31.11)	26.55 (23.23, 31.10)	26.94 (24.47, 31.25)	31.52 (25.64, 38.40)	30.40 (26.99, 36.03)	28.93 (25.10, 33.66)	28.40 (25.14, 32.43)	<0.001
Wasit (cm)	97.10 (86.70, 108.40)	94.38 (82.20, 107.90)	93.00 (82.40, 106.70)	93.40 (83.50, 104.60)	93.70 (86.01, 105.24)	107.67 (95.59, 124.34)	106.28 (96.40, 117.42)	100.60 (90.75, 111.40)	99.50 (90.60, 108.60)	<0.001
Heart rate (bpm/min)	72.0 (64.0, 80.0)	70.0 (62.0, 80.0)	70.0 (62.0, 78.0)	72.0 (64.0, 78.0)	70.0 (62.0, 77.0)	70.5 (66.0, 83.6)	72.0 (64.0, 82.0)	72.0 (64.0, 80.0)	72.0 (64.0, 80.0)	0.047
SBP (mmHg)	119.0 (110.0, 130.0)	118.0 (108.0, 130.0)	116.0 (108.0, 128.0)	116.0 (108.0, 127.4)	116.0 (108.0, 126.0)	125.4 (118.1, 138.2)	126.0 (114.3, 138.0)	122.0 (112.0, 134.0)	122.0 (112.0, 132.0)	<0.001
DBP (mmHg)	70.0 (62.0, 78.0)	66.0 (58.0, 74.0)	68.0 (60.0, 74.0)	70.0 (62.0, 76.0)	68.0 (60.6, 77.7)	66.6 (59.9, 78.1)	72.0 (64.0, 82.0)	72.0 (64.0, 78.0)	72.0 (66.0, 80.0)	<0.001
Smoking status										0.002
Never	8,702.0 (55.1%)	799.0 (53.3%)	2,308.0 (56.5%)	1,285.0 (59.7%)	69.0 (52.1%)	6.0 (16.5%)	234.0 (52.5%)	1,517.0 (51.9%)	2,484.0 (54.9%)	
Former	3,593.0 (24.5%)	363.0 (25.3%)	855.0 (23.4%)	451.0 (22.0%)	28.0 (23.3%)	7.0 (36.9%)	141.0 (28.8%)	738.0 (27.9%)	1,010.0 (23.9%)	
Current	3,085.0 (20.3%)	305.0 (21.4%)	787.0 (20.2%)	385.0 (18.2%)	25.0 (24.6%)	10.0 (46.5%)	86.0 (18.8%)	566.0 (20.2%)	921.0 (21.2%)	
Drinking status										0.085
Never	5,246.0 (27.9%)	551.0 (29.5%)	1,366.0 (28.7%)	681.0 (25.7%)	33.0 (22.3%)	8.0 (28.8%)	177.0 (34.3%)	937.0 (26.5%)	1,493.0 (28.1%)	
Light	5,373.0 (35.6%)	470.0 (34.4%)	1,377.0 (34.6%)	753.0 (35.2%)	45.0 (37.9%)	9.0 (36.4%)	157.0 (37.2%)	1,047.0 (37.7%)	1,515.0 (35.3%)	
Moderate	4,540.0 (35.1%)	429.0 (34.8%)	1,148.0 (35.1%)	661.0 (38.1%)	41.0 (36.7%)	5.0 (26.1%)	119.0 (26.5%)	794.0 (34.2%)	1,343.0 (35.2%)	
Heavy	221.0 (1.5%)	17.0 (1.2%)	59.0 (1.6%)	26.0 (1.1%)	3.0 (3.1%)	1.0 (8.7%)	8.0 (2.1%)	43.0 (1.6%)	64.0 (1.4%)	
TC (mg/L)	189.00 (164.00, 217.00)	134.00 (121.00, 147.00)	160.00 (149.00, 171.00)	184.00 (174.00, 197.00)	219.00 (202.00, 231.00)	163.07 (153.66, 173.78)	176.00 (167.00, 188.00)	195.00 (185.00, 206.00)	232.00 (215.00, 253.00)	<0.001
TG (mg/L)	102.00 (71.00, 149.00)	84.00 (54.03, 133.00)	81.00 (59.00, 117.00)	79.00 (61.00, 101.00)	75.00 (58.00, 102.76)	298.00 (263.11, 369.52)	204.00 (157.12, 261.00)	126.00 (93.00, 178.00)	121.00 (90.00, 169.00)	<0.001
LDL-C (mg/L)	111.00 (89.00, 135.00)	60.00 (53.00, 65.00)	86.00 (78.00, 93.00)	109.00 (104.00, 115.00)	134.53 (131.00, 140.97)	64.98 (60.67, 67.00)	92.00 (86.00, 96.00)	118.00 (111.00, 124.00)	149.00 (138.00, 167.00)	<0.001
HDL-C (mg/L)	52.00 (43.00, 63.00)	52.00 (42.00, 65.00)	54.00 (45.00, 65.00)	57.00 (48.00, 69.00)	61.00 (50.00, 72.00)	39.33 (33.13, 43.18)	43.00 (34.00, 52.00)	48.00 (40.00, 59.00)	52.00 (43.00, 62.00)	<0.001
APOB (mg/dL)	90.0 (74.0, 107.0)	57.0 (49.0, 64.0)	72.0 (65.0, 78.0)	83.0 (78.0, 86.0)	87.0 (83.0, 88.0)	95.0 (92.0, 98.0)	95.0 (92.0, 101.0)	98.0 (94.0, 105.0)	116.0 (106.0, 129.0)	<0.001
Non-HDL cholesterol (mg/dL)	134.00 (109.00, 161.00)	77.00 (69.00, 86.00)	104.00 (95.00, 112.00)	127.00 (120.00, 134.00)	151.00 (146.00, 157.00)	123.84 (114.85, 131.25)	132.00 (122.00, 143.00)	144.00 (135.00, 153.00)	177.00 (162.00, 198.00)	<0.001
AST (U/L)	23.00 (19.00, 27.00)	23.00 (19.00, 28.00)	22.00 (19.00, 27.00)	22.00 (19.00, 26.00)	22.00 (18.18, 26.00)	28.19 (17.00, 30.01)	24.00 (20.00, 29.00)	23.00 (20.00, 28.00)	23.00 (20.00, 28.00)	<0.001
ALT(U/L)	21.00 (16.00, 28.00)	20.00 (15.00, 27.00)	19.00 (15.00, 26.00)	20.00 (16.00, 26.00)	20.00 (16.00, 26.00)	27.38 (21.43, 32.50)	23.85 (19.00, 33.00)	22.00 (17.00, 30.00)	22.00 (17.00, 31.00)	<0.001
Cr (mg/dL)	0.84 (0.72, 0.99)	0.88 (0.73, 1.03)	0.82 (0.70, 0.99)	0.82 (0.70, 0.95)	0.81 (0.67, 0.95)	0.81 (0.74, 0.97)	0.83 (0.71, 1.00)	0.87 (0.73, 1.00)	0.85 (0.73, 0.99)	<0.001
Ca (mmol/L)	2.35 (2.30, 2.40)	2.33 (2.28, 2.40)	2.33 (2.28, 2.40)	2.35 (2.30, 2.40)	2.33 (2.28, 2.38)	2.33 (2.29, 2.39)	2.35 (2.30, 2.40)	2.35 (2.28, 2.40)	2.35 (2.30, 2.40)	<0.001
25OHD3 (nmol/L)	65.80 (49.50, 83.40)	63.30 (47.10, 82.80)	66.10 (50.20, 85.20)	66.90 (49.60, 84.48)	68.54 (50.07, 83.39)	64.01 (53.46, 74.24)	64.10 (46.42, 79.40)	64.10 (48.80, 81.04)	66.30 (49.50, 83.30)	0.036
Diabetes (*n*, %)										<0.001
Yes	1,786.0 (9.0%)	343.0 (19.8%)	488.0 (9.7%)	136.0 (4.4%)	2.0 (1.5%)	7.0 (38.2%)	113.0 (26.5%)	329.0 (9.1%)	368.0 (5.8%)	
No	13,283.0 (88.9%)	1,100.0 (78.5%)	3,386.0 (88.4%)	1,953.0 (94.0%)	119.0 (98.1%)	16.0 (61.8%)	333.0 (70.9%)	2,426.0 (88.6%)	3,950.0 (91.9%)	
Borderline	311.0 (2.1%)	24.0 (1.7%)	76.0 (1.9%)	32.0 (1.7%)	1.0 (0.4%)	0.0 (0.0%)	15.0 (2.6%)	66.0 (2.3%)	97.0 (2.3%)	
Hypertension (*n*, %)	5,294.0 (32.1%)	593.0 (38.0%)	1,297.0 (29.9%)	582.0 (25.1%)	36.0 (23.1%)	10.0 (68.2%)	232.0 (53.7%)	1,067.0 (34.9%)	1,477.0 (31.9%)	<0.001
Heart failure (*n*, %)	579.0 (2.9%)	122.0 (6.3%)	170.0 (3.7%)	40.0 (1.2%)	3.0 (2.3%)	1.0 (7.9%)	31.0 (7.0%)	97.0 (2.6%)	115.0 (1.9%)	<0.001
CAD (*n*, %)	769.0 (4.1%)	203.0 (12.4%)	269.0 (5.8%)	41.0 (1.4%)	1.0 (1.4%)	4.0 (25.0%)	52.0 (9.6%)	106.0 (3.4%)	93.0 (1.6%)	<0.001
Stroke (*n*, %)	665.0 (3.3%)	132.0 (6.5%)	196.0 (3.9%)	52.0 (1.7%)	4.0 (4.7%)	4.0 (16.3%)	22.0 (4.2%)	114.0 (3.1%)	141.0 (2.6%)	<0.001
Emphysema (*n*, %)	313.0 (1.9%)	40.0 (3.1%)	90.0 (2.1%)	33.0 (1.4%)	2.0 (0.8%)	1.0 (6.2%)	12.0 (2.4%)	58.0 (1.9%)	77.0 (1.7%)	0.062
Chronic bronchitis (*n*, %)	913.0 (5.9%)	101.0 (5.5%)	249.0 (6.3%)	97.0 (4.6%)	8.0 (5.1%)	4.0 (19.1%)	28.0 (7.3%)	173.0 (5.6%)	253.0 (6.3%)	0.100
Cancer (*n*, %)	1,436.0 (9.8%)	152.0 (10.4%)	387.0 (10.0%)	153.0 (7.8%)	13.0 (9.6%)	4.0 (22.9%)	56.0 (12.1%)	305.0 (10.8%)	366.0 (9.7%)	0.100
Statins (*n*, %)	2,737.0 (17.3%)	530.0 (36.3%)	913.0 (22.9%)	215.0 (10.1%)	3.0 (1.7%)	10.0 (59.8%)	181.0 (43.8%)	519.0 (18.4%)	366.0 (7.4%)	<0.001
TG-lowering medications	241.0 (1.7%)	33.0 (2.8%)	71.0 (1.9%)	11.0 (0.4%)	0.0 (0.0%)	1.0 (7.9%)	19.0 (5.2%)	55.0 (2.3%)	51.0 (1.1%)	<0.001
Cholesterol-lowering medications	240.0 (1.7%)	58.0 (4.1%)	68.0 (1.9%)	10.0 (0.5%)	0.0 (0.0%)	2.0 (16.2%)	19.0 (4.1%)	33.0 (1.5%)	50.0 (1.2%)	<0.001
All-cause of mortality (*n*, %)	1,771.0 (8.8%)	280.0 (16.6%)	475.0 (9.1%)	160.0 (5.4%)	14.0 (10.2%)	3.0 (10.8%)	74.0 (12.1%)	317.0 (8.9%)	448.0 (7.5%)	<0.001
Cardivascular mortality (*n*, %)	443.0 (2.1%)	76.0 (4.4%)	121.0 (2.2%)	34.0 (0.8%)	2.0 (0.4%)	1.0 (0.9%)	21.0 (4.0%)	82.0 (2.2%)	106.0 (1.7%)	<0.001
Cerebrovascular mortality (*n*, %)	109.0 (0.5%)	18.0 (1.2%)	24.0 (0.5%)	9.0 (0.2%)	0.0 (0.0%)	0.0 (0.0%)	3.0 (0.4%)	20.0 (0.5%)	35.0 (0.5%)	0.009
Following-up time (month)	101.0 (67.0, 137.0)	88.0 (60.0, 128.0)	98.0 (65.0, 134.0)	100.0 (73.0, 130.0)	96.4 (83.5, 121.0)	121.8 (83.8, 152.1)	105.0 (70.0, 156.4)	103.0 (64.0, 148.0)	104.0 (69.0, 139.0)	<0.001

Numbers of participants were unweighted, and all percentage estimates are weighted. Other data are weighted estimates, and expressed as median (percentile 25, percentile 75).

BMI: body mass index, SBP: systolic blood pressure, DBP: diastolic blood pressure, TC: total cholesterol, TG: triglyceride, LDL-C: low-density lipoprotein-cholesterol, HDL-C: high-density lipoprotein-cholesterol, AST: aspartate transaminase, ALT: alanine transaminase, Cr: creatinine, CAD: coronary artery disease. *P value < 0.001 indicates that the observed differences between groups are statistically significant at a very high level of confidence.

### Independent associations of LDL-cholesterol and ApoB with mortality

**Figure 2.**
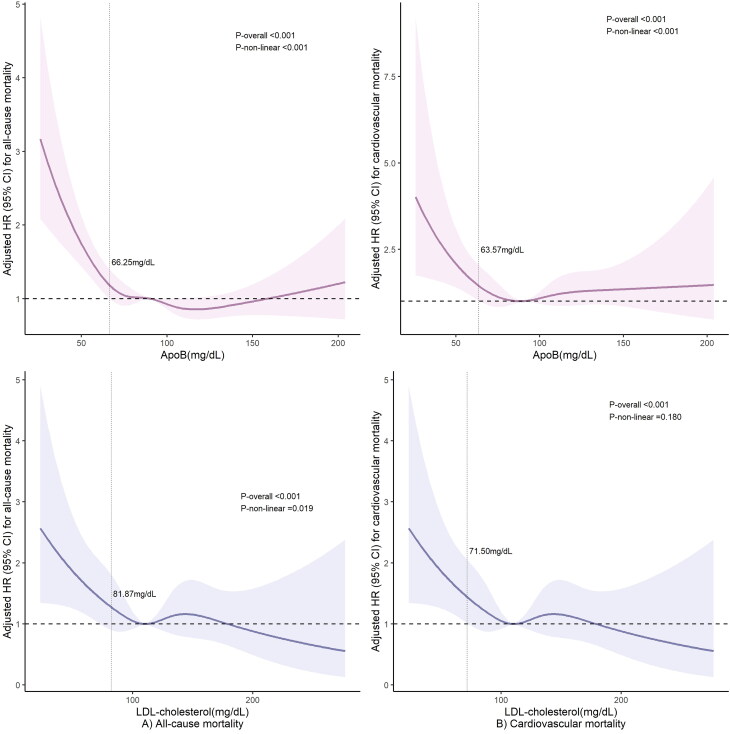
Restricted cubic spline curves of the risk for all-cause (*A*) and cardiovascular (*B*) mortality according to the dose-response associations between apoB and LDL-cholesterol. Restricted cubic splines were constructed with five knots located at the 5th, 27.5th, 50th, 72.5th, and 95th percentiles of each exposure. Adjusted hazard ratios (95%CI) were calculated with adjustments for age, sex, ethnicity, marital status, education status, BMI, smoking status, systolic and diastolic blood pressure, history of diabetes, hypertension, heart failure, CHD, stroke, emphysema, bronchitis, cancer, use of statins, cholesterol-lowering medications (except statins), and triglyceride-lowering medications.

A total of 1,771 (8.8%) all-cause mortality events occurred over a median follow-up of 101.0 months (IQR: 67–137 months), with 443 (2.1%) deaths attributed to cardiovascular disease and 109 (0.5%) to cerebrovascular disease. In multivariable-adjusted analyses, low LDL-cholesterol (<70 mg/dL) was associated with increased risks of all-cause (HR 1.66, 95% CI 1.33–2.06) and cardiovascular mortality (HR 1.65, 95% CI 1.12–2.43), but not cerebrovascular mortality (HR 2.13, 95% CI 1.01–4.49). Low apoB (<90 mg/dL) was linked to higher all-cause mortality risk (HR 0.79, 95% CI 0.69–0.89) but not cardiovascular or cerebrovascular mortality (Table S1). Restricted cubic spline analysis indicated a non-linear relationship between LDL-cholesterol and all-cause mortality (*P* for non-linearity <0.05; Figure 2A) and a linear association with cardiovascular mortality (*P* for non-linearity >0.05; Figure 2B). Moreover, apoB exhibited non-linear dose-response patterns in relation to all-cause and cardiovascular mortality risks (all *P* for overall <0.001; all *P* for non-linearity <0.001; Figure 2A and 2B).

### Combined associations of LDL-cholesterol and apoB with mortality

The analysis first focused on individuals with apoB <90 mg/dL. Within this group, those with LDL-cholesterol <100 mg/dL exhibited a higher mortality risk compared to the reference group (LDL-cholesterol 100–129 mg/dL). The HR for all-cause mortality was 2.07 (95% CI: 1.55–2.77) in the LDL-cholesterol <70 mg/dL group and 1.59 (95% CI: 1.37–1.78) in the 70–99 mg/dL group. Additionally, the HR for cardiovascular mortality reached 2.42 (95% CI: 1.37–4.29) in individuals with LDL-cholesterol <70 mg/dL. In contrast, among participants with apoB ≥90 mg/dL and LDL-cholesterol <100 mg/dL, no significant increase in all-cause or cardiovascular mortality was observed (Table 2).

**Table 2.** Associations of LDL-cholesterol with all-cause, cardiovascular disease mortality stratified by the ApoB median.

All-cause	LDL-C	Crude model			Model 1			Model 2		
mortality	(mg/dL)	HR	95% CI	*P*-value	HR	95% CI	*P*-value	HR	95% CI	*P*-value
ApoB < 90 (mg/dL)	100–129	Ref.			Ref.			Ref.		
	<70	3.37	2.64–4.29	<0.001	2.19	1.70–2.81	<0.001	2.07	1.55–2.77	<0.001
	70–99	1.74	1.41–2.15	<0.001	1.40	1.11–1.77	0.004	1.37	1.06–1.78	0.017
	≥130	1.93	0.93–4.02	0.079	1.54	0.75–3.17	0.238	1.32	0.64–2.71	0.445
ApoB ≥ 90 (mg/dL)	100–129	Ref.			Ref.			Ref.		
	<70	1.03	0.22–4.86	0.968	0.64	0.13–3.21	0.590	0.27	0.04–1.85	0.184
	70–99	1.29	0.91–1.82	0.153	1.08	0.80–1.46	0.610	0.87	0.62–1.22	0.415
	≥130	0.85	0.71–1.02	0.088	0.94	0.79–1.11	0.454	0.95	0.79–1.14	0.570
Cardiovascular mortality										
ApoB < 90 (mg/dL)	100–129	Ref.			Ref.			Ref.		
	<70	5.70	3.51–9.27	<0.001	3.25	2.03–5.19	<0.001	2.42	1.37–4.29	0.002
	70–99	2.71	1.78–4.13	<0.001	1.96	1.29–2.97	0.002	1.59	0.96–2.63	0.072
	≥130	0.48	0.11–2.05	0.319	0.36	0.09–1.52	0.164	0.34	0.08–1.37	0.128
ApoB ≥ 90 (mg/dL)	100–129	Ref.			Ref.			Ref.		
	<70	0.37	0.05–2.97	0.350	0.23	0.03–2.01	0.186	0.11	0.01–1.16	0.066
	70–99	1.72	0.91–3.26	0.095	1.42	0.79–2.58	0.243	1.04	0.56–1.95	0.890
	≥130	0.78	0.54–1.13	0.192	0.88	0.60–1.29	0.498	0.94	0.61–1.43	0.757

**Figure 3.**
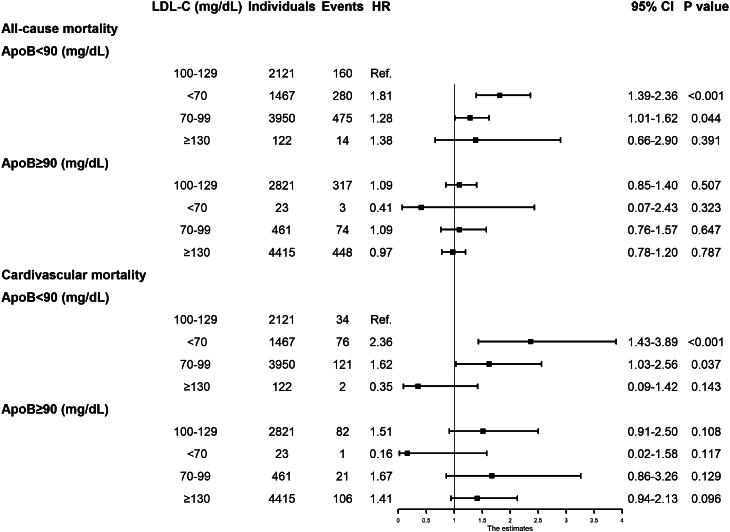
Combined association of apoB and LDL-cholesterol levels with all-cause and cardiovascular disease mortality. The multivariable cox model was adjusted for age, sex, ethnicity, marital status, education status, BMI, smoking status, systolic and diastolic blood pressure, history of diabetes, hypertension, heart failure, CHD, stroke, emphysema, chronic bronchitis, cancer, use of statins, cholesterol-lowering medications (except statins), and triglyceride-lowering medications.

Figure 3 presents the combined impact of apoB and LDL-cholesterol levels on mortality. The highest mortality risk was identified in individuals with apoB <90 mg/dL and LDL-cholesterol <70 mg/dL. The combination of apoB <90 mg/dL with LDL-cholesterol <70 mg/dL (HR: 1.81; 95% CI: 1.39–2.36) and 70–99 mg/dL (HR: 1.28; 95% CI: 1.01–1.62) demonstrated the strongest association with all-cause mortality. These results suggest that lower apoB levels increase the mortality risks linked to reduced LDL-cholesterol levels.

## Discussion

This study provides new insights into the association between apoB, LDL-cholesterol levels, and mortality risk in a large sample of U.S. adults. Low apoB in combination with low LDL-cholesterol levels was linked to an increased risk of all-cause and cardiovascular mortality. In contrast, no such association was observed between high apoB and low LDL-cholesterol levels. LDL-cholesterol levels <70 mg/dL were positively associated with the highest mortality risk in participants with apoB <90 mg/dL.

In this study, low LDL-cholesterol levels were associated with an elevated risk of all-cause, cardiovascular and cerebrovascular mortality. While most existing studies have emphasized the benefits of lowering LDL-cholesterol, the potential risks of very low LDL-cholesterol levels warrant attention. Several studies have proposed a link between low LDL-cholesterol and increased all-cause mortality. Johannesen et al. identified such an association in the Danish population through a prospective cohort study, with an HR of 1.25 (95% CI 1.15 to 1.36) [30]. While Wen et al. found that lower serum LDL-cholesterol levels independently predicted higher mortality following acute ICH [31]. A Mendelian randomization analysis indicated that individuals with LDL-cholesterol levels below the recommended threshold of 70 mg/dL had a heightened risk of adverse outcomes in British and Chinese populations [32]. Additionally, prospective cohort studies have shown that reduced LDL-cholesterol levels correlate with increased all-cause mortality risk in middle-aged and elderly populations in China and the US [29,33]. Conversely, a Chinese longitudinal health survey revealed that higher LDL-cholesterol levels were associated with a lower risk of all-cause mortality among the elderly, with each 1 mmol/L increase in LDL-cholesterol concentration corresponding to a 19% reduction in 3-year all-cause mortality (HR 0.81, 95% CI 0.71–0.92) [34].

Nonetheless, several studies have examined the association between low LDL-cholesterol levels and all-cause mortality risk, reporting no correlation with apoB levels. Retention of apoB-containing lipoproteins within the arterial wall serves as a key initiating and propagating factor in atherogenesis [18]. Furthermore, these prospective cohort analyses demonstrated that apoB serves as a more precise indicator of mortality risk compared to LDL-cholesterol in statin-treated patients [22,35]. Ference et al. reported that the clinical benefit of reducing triglyceride and LDL-cholesterol levels was likely proportional to the absolute decline in apoB, based on data from 654,783 participants in North America and Europe [36]. A Mendelian randomization analysis further suggested that the effectiveness of LDL-cholesterol reduction may rely on the extent of apoB-containing lipoprotein particle reduction [25]. Accordingly, apoB plays a central role in assessing the relationship between LDL-cholesterol levels and mortality. The present analysis identified a significant association between low LDL-cholesterol and increased all-cause and cardiovascular mortality risk in populations with low apoB levels, whereas no such relationship was observed in those with high apoB levels. In our study, the lack of association between high LDL-cholesterol (>130 mg/dL) and mortality may be attributed to the unique demographic and clinical characteristics of our cohort, including the distribution of apoB levels, the prevalence of statin use, and the low baseline cardiovascular risk. Additionally, variations in statistical power or unmeasured confounding factors could contribute to these divergent results. Further studies are needed to validate these findings and elucidate the underlying mechanisms.

Lower LDL-cholesterol levels may be linked to an increased risk of death from infection [10], cancer [17], and ICH [16,37,38], thereby contributing to a higher overall risk of mortality. Furthermore, elevated apoB levels are associated with a reduced risk of ICH [39]. Therefore, monitoring the potentially harmful effects of very low LDL-cholesterol levels in individuals with low apoB levels is essential. These results highlight the adverse outcomes associated with extremely low LDL-cholesterol and apoB levels, offering a novel perspective for lipid management in clinical practice.

However, this study presents some limitations. First, due to its observational design, causal relationships between LDL-cholesterol levels and mortality risk cannot be established. Second, LDL-cholesterol and apoB levels are only measured at baseline, and a single measurement may not accurately reflect average levels over the long follow-up period. Third, some subgroups in our analysis, particularly those with extreme LDL-C or apoB levels, had small sample sizes. Although these groups were retained to preserve the granularity of the data, the findings from these subgroups did not reach statistical significance and do not affect the main conclusions of our study. Finally, although we adjusted for major confounders, the possibility of residual confounding due to unmeasured or uncontrolled factors (e.g. family history of cardiovascular disease, statin medication adherence) cannot be entirely ruled out.

## Conclusions

This study demonstrates that LDL-cholesterol levels <100 mg/dL are linked to a higher mortality risk in individuals with apoB <90 mg/dL, but no such association is observed in those with apoB ≥90 mg/dL. Thus, low apoB levels combined with low LDL-cholesterol levels are associated with increased risks of all-cause and cardiovascular mortality, highlighting the significance of lipid profile management in the general population.

## Supplementary Material

Table Supplemental 1.docx

## Data Availability

The data that support the findings of this study are available from the corresponding author on reasonable request.
