# Supplementary material for: Low apolipoprotein B and LDL-cholesterol are associated with the risk of cardiovascular and all-cause mortality: a prospective cohort
Source: Ann Med. 2025 Jul 11;57(1):2529565. doi: 10.1080/07853890.2025.2529565 (PMC12258213; doi:10.1080/07853890.2025.2529565)
Supplement: Table Supplemental 1.docx [file IANN_A_2529565_SM2743.docx]

**Table S1 Associations of LDL-cholesterol and apoB levels with all-cause, cardiovascular and cerebrovascular disease mortality**

|  |  | Crude model | | | Model 1 | | | Model 2 | | |
| --- | --- | --- | --- | --- | --- | --- | --- | --- | --- | --- |
| All-cause mortality | Value | **HR** | **95% CI** | **P value** | **HR** | **95% CI** | **P value** | **HR** | **95% CI** | **P value** |
| ApoB(mg/dL) | <90 | Ref. |  |  | Ref. |  |  | Ref. |  |  |
|  | ≥90 | 0.83 | 0.74-0.93 | 0.002 | 0.74 | 0.65-0.83 | <0.001 | 0.79 | 0.69-0.89 | <0.001 |
| LDL-C (mg/dL) | 100-129 | Ref. |  |  | Ref. |  |  | Ref. |  |  |
|  | <70 | 2.49 | 2.07-2.99 | <0.001 | 1.96 | 1.61-2.39 | <0.001 | 1.66 | 1.33-2.06 | <0.001 |
|  | 70-99 | 1.33 | 1.15-1.53 | <0.001 | 1.27 | 1.10-1.47 | 0.002 | 1.18 | 1.00-1.39 | 0.045 |
|  | ≥130 | 1.03 | 0.89-1.19 | 0.73 | 0.98 | 0.85-1.13 | 0.758 | 0.93 | 0.79-1.09 | 0.355 |
| Cardiovascular mortality |  |  |  |  |  |  |  |  |  |  |
| ApoB(mg/dL) | <90 | Ref. |  |  | Ref. |  |  | Ref. |  |  |
|  | ≥90 | 0.86 | 0.69-1.07 | 0.183 | 0.79 | 0.63-0.99 | 0.037 | 0.92 | 0.73-1.16 | 0.488 |
| LDL-C (mg/dL) | 100-129 | Ref. |  |  | Ref. |  |  | Ref. |  |  |
|  | <70 | 3.03 | 2.16-4.25 | <0.001 | 2.25 | 1.60-3.16 | <0.001 | 1.65 | 1.12-2.43 | 0.010 |
|  | 70-99 | 1.57 | 1.14-2.16 | 0.005 | 1.44 | 1.06-1.97 | 0.021 | 1.2 | 0.85-1.69 | 0.295 |
|  | ≥130 | 1.03 | 0.75-1.40 | 0.863 | 0.99 | 0.73-1.36 | 0.966 | 1.03 | 0.75-1.41 | 0.874 |
| Cerebrovascular mortality |  |  |  |  |  |  |  |  |  |  |
| ApoB(mg/dL) | <90 | Ref. |  |  | Ref. |  |  | Ref. |  |  |
|  | ≥90 | 0.84 | 0.52-1.37 | 0.491 | 0.8 | 0.49-1.30 | 0.369 | 0.9 | 0.55-1.47 | 0.665 |
| LDL-C (mg/dL) | 100-129 | Ref. |  |  | Ref. |  |  | Ref. |  |  |
|  | <70 | 3.76 | 1.89-7.49 | <0.001 | 2.58 | 1.29-5.15 | 0.007 | 2.13 | 1.01-4.49 | 0.047 |
|  | 70-99 | 1.28 | 0.69-2.39 | 0.429 | 1.13 | 0.60-2.16 | 0.701 | 1.04 | 0.52-2.09 | 0.915 |
|  | ≥130 | 1.21 | 0.73-2.03 | 0.461 | 1.21 | 0.73-2.00 | 0.457 | 1.14 | 0.67-1.94 | 0.635 |

Model 1, adjusting for age and sex

Model 2, adjusting for Model 1 plus body mass index(BMI), race, marital status, education status, smoking and drinking status, systolic blood pressure, diastolic blood pressure, history of diabetes, hypertension, heart failure, coronary heart disease, stroke, emphysema, chronic bronchitis, cancer, use of statins, other cholesterol-lowering medications, and triglyceride-lowering medications
